# Generation of Human Immunosuppressive Myeloid Cell Populations in Human Interleukin-6 Transgenic NOG Mice

**DOI:** 10.3389/fimmu.2018.00152

**Published:** 2018-02-02

**Authors:** Asami Hanazawa, Ryoji Ito, Ikumi Katano, Kenji Kawai, Motohito Goto, Hiroshi Suemizu, Yutaka Kawakami, Mamoru Ito, Takeshi Takahashi

**Affiliations:** ^1^Laboratory Animal Research Department, Central Institute for Experimental Animals (CIEA), Kawasaki, Japan; ^2^Pathological Analysis Center, Central Institute for Experimental Animals (CIEA), Kawasaki, Japan; ^3^Animal Resources Center, Central Institute for Experimental Animals (CIEA), Kawasaki, Japan; ^4^Division of Cellular Signaling, Institute for Advanced Medical Research, Keio University School of Medicine, Tokyo, Japan

**Keywords:** humanized mice, MDSCs, NOG-hIL-6 Tg mice, TAMs, tumor microenvironment

## Abstract

The tumor microenvironment contains unique immune cells, termed myeloid-derived suppressor cells (MDSCs), and tumor-associated macrophages (TAMs) that suppress host anti-tumor immunity and promote tumor angiogenesis and metastasis. Although these cells are considered a key target of cancer immune therapy, *in vivo* animal models allowing differentiation of human immunosuppressive myeloid cells have yet to be established, hampering the development of novel cancer therapies. In this study, we established a novel humanized transgenic (Tg) mouse strain, human interleukin (hIL)-6-expressing NOG mice (NOG-hIL-6 transgenic mice). After transplantation of human hematopoietic stem cells (HSCs), the HSC-transplanted NOG-hIL-6 Tg mice (HSC-NOG-hIL-6 Tg mice) showed enhanced human monocyte/macrophage differentiation. A significant number of human monocytes were negative for HLA-DR expression and resembled immature myeloid cells in the spleen and peripheral blood from HSC-NOG-hIL-6 Tg mice, but not from HSC-NOG non-Tg mice. Engraftment of HSC4 cells, a human head and neck squamous cell carcinoma-derived cell line producing various factors including IL-6, IL-1β, macrophage colony-stimulating factor (M-CSF), and vascular endothelial growth factor (VEGF), into HSC-NOG-hIL-6 Tg mice induced a significant number of TAM-like cells, but few were induced in HSC-NOG non-Tg mice. The tumor-infiltrating macrophages in HSC-NOG-hIL-6 Tg mice expressed a high level of CD163, a marker of immunoregulatory myeloid cells, and produced immunosuppressive molecules such as arginase-1 (Arg-1), IL-10, and VEGF. Such cells from HSC-NOG-hIL-6 Tg mice, but not HSC-NOG non-Tg mice, suppressed human T cell proliferation in response to antigen stimulation in *in vitro* cultures. These results suggest that functional human TAMs can be developed in NOG-hIL-6 Tg mice. This mouse model will contribute to the development of novel cancer immune therapies targeting immunoregulatory/immunosuppressive myeloid cells.

## Introduction

Humanized mouse technology has enabled reconstitution of human hematopoietic and immune systems in immunodeficient mice (1, 2). Accumulating evidence suggests that this novel technology is suitable for studying several infectious diseases including human immunodeficiency virus (HIV), Epstein-Barr virus, and malaria (3–6). These studies have revealed that replication of pathogens is possible *in vivo*, and that immune responses against these pathogens are elicited, even if in a limited manner. Thus, humanized mice are useful instruments for studying *in vivo* human physiology and conducting preclinical studies for novel drugs. In this context, the use of humanized mice has been applied in immuno-oncological studies to evaluate drug efficiencies (7, 8). Considering the complex pathology of tumors, it is important to clarify which cellular lineages contribute to tumor formation and disease progression, and whether those cells are present in humanized mice (9).

Humanized mice are usually produced using extremely severe immunodeficient mouse strains including, NOD/shi-scid/IL-2Rγ^null^ (NOG), NOD/LtSz-scid/IL-2Rγ^null^ (NSG), or BALB/c-Rag2^null^/IL-2Rγ^null^ (BRG). Human immune systems can be reconstituted in these mice by transplanting human CD34^+^ hematopoietic stem cells (HSCs) (10–12). Humanized mice based on these platform strains harbor limited human myeloid cell lineages including granulocytes, monocytes, macrophages, and their progenitors. As several of these cell lineages are relevant to disease development, our group and others have genetically modified these platform strains by introducing human cytokine genes to improve myeloid differentiation. For example, myelopoiesis was markedly enhanced in NOG-human (h) granulocyte macrophage colony-stimulating factor (GM-CSF)/interleukin (IL)-3 Tg mice (NOG-hGM/3 Tg) compared to parental NOG mice, and mast cells that developed in this strain were fully functional in mediating passive cutaneous anaphylaxis (PCA) (13). Similar results were obtained in NSG mice with human GM-CSF/IL-3/stem cell factor transgenes (NSG-SGM3). NSG-SGM3 mice showed enhanced differentiation of human myeloid lineage cells (14). BLT (bone marrow–liver–thymus) mice on the NSG-SGM3 background, a type of humanized mice generated by engrafting human fetal-derived thymus and liver in renal capsule and subsequent HSC transplantation, induced human PCA and passive systemic anaphylaxis mediated by human mast cells (15). BRG mice have been modified to generate MITRG mice, in which the murine macrophage colony-stimulating factor (M-CSF), IL-3, GM-CSF, and thrombopoietin genes were replaced by the human homologs, and MISTRG mice, which also contain the human signal-regulatory protein alpha gene (16). The development of functional human monocytes, macrophages, and natural killer (NK) cells has been promoted in these mice. For example, ~3-fold high number of CD33^+^ total myeloid cells developed in NOG-hGM/3 Tg compared to NOG mice (13), ~3-fold increase of CD33^+^ cells in frequency in NSG-SGM3 (15), and ~10-fold CD33^+^ cells in MITRG compared to NSG mice (16). In addition, human NK cells consisted of 10–20% of mononuclear cells (MNCs) in peripheral blood in MISTRG mice (16). Furthermore, human macrophages infiltrate a human tumor xenograft in MITRG or MISTRG mice (16). These results suggest that human myeloid cell development can be induced in humanized mice by introducing the appropriate human cytokines.

The tumor microenvironment consists of an unusual variety of cell types that include not only cancer cells but also fibroblasts, endothelial cells in blood vessels and lymph ducts, and immune cells such as lymphocytes and myeloid cells. Patients with cancer and tumor masses have increased numbers of cells that phenotypically resemble immature myeloid cells, and the prognosis of these patients is inversely correlated with the number of these immature myeloid cells. Thus, immunoregulatory activity can facilitate tumor progression by preventing host immune systems from attacking a tumor and by inducing factors that promote angiogenesis (17). Tumor-associated macrophages (TAMs) and myeloid-derived supressor cells (MDSCs), especially, are two representatives of such immunosuppressive myeloid cells. TAMs produce various types of immunosuppressive molecules including arginase-1 (Arg-1), IL-10, tumor growth factor-β, or prostaglandin E_2_ (PGE_2_); and factors related to angiogenesis or cell proliferation such as vascular endothelial growth factor (VEGF), IL-8, basic fibroblast growth factor (bFGF), hepatocyte growth factor, epidermal growth factor, or platelet-derived growth factor (18, 19). MDSCs also produce Arg-1, inducible nitric oxide synthase, reactive oxygen species, and peroxynitrite for immunosuppression (20). Studies investigating the molecular mechanisms in the induction of TAMs and MDSCs revealed the critical role of inflammatory cytokines. IL-6, in particular, plays an essential role in the induction, as IL-4 receptor alpha chain (IL-4Rα)^+^ MDSCs are produced in *in vitro* cultures of mouse bone marrow (BM) cells incubated with IL-6, granulocyte colony-stimulating factor (G-CSF), and GM-CSF (21). In addition, IL-6 promotes differentiation of granulocytic MDSCs (22, 23), and the number of monocytic MDSCs increases when IL-6 production is enhanced due to infection by hepatitis B virus (24). These studies suggest that human IL-6 (hIL-6) is an indispensable requirement for recapitulating the human tumor microenvironment in humanized mice.

In this study, we established a novel NOG sub-strain, NOG-hIL-6 Tg mice. We demonstrated that after transplantation of human HSCs, a significantly higher numbers of human monocytes and macrophages were induced in NOG-hIL-6 Tg mice than in NOG non-Tg mice. We further demonstrated that after tumor engraftment, significant numbers of immature myeloid cells, phenotypically resembling TAMs in clinical patients, differentiated in HSC transplanted NOG-hIL-6 Tg mice (HSC-NOG-hIL-6 Tg mice), whereas few myeloid cells were observed in HSC-NOG non-Tg mice. This novel mouse model is a unique tool for studying the pathology of tumor formation and will facilitate drug discovery targeting TAMs.

## Materials and Methods

### Mice

NOD/ShiJic/scid/IL-2Rγ*^null^* (NOG) mice and NOD/ShiJic (NOD) mice were used in this study. To generate hIL-6-expressing Tg NOG mice, a DNA fragment containing hIL-6 cDNA, under the control of the cytomegalovirus (CMV) promoter, was microinjected into female NOD mouse eggs fertilized by male NOG mice. A founder mouse was backcrossed with NOG mice to obtain NOG-hIL-6 Tg mice (NOD.Cg-*prkdc^scid^il2rγ^tm1Sug^*/ShiJic CMV-IL-6 Tg). Serum levels of hIL-6 were measured using hIL-6 Quantikine enzyme-linked immunosorbent assay (ELISA) kits (R&D systems, Minneapolis, MN, USA). All of the mice were maintained in the Central Institute for Experimental Animals (CIEA) under specific pathogen-free conditions.

### Transplantation of Human HSCs

Human umbilical-cord-blood-derived CD34^+^ HSCs were purchased from Allcells (Alameda, CA, USA). For transplantation, 6–12-week-old adult mice were irradiated (2.5 Gy) (MBR-1505R; Hitachi Medical, Tokyo, Japan), and 5 × 10^4^ HSCs were injected intravenously within 24 h. To monitor human hematopoiesis, the mice were bled every 3 weeks over a total of 3 months, and the MNCs were analyzed by flow cytometry.

### Cell Lines

A human tumor cell line, HSC4, derived from human head and neck squamous cell carcinoma, was provided by Y. Kawakami (Keio University School of Medicine, Tokyo, Japan). HSC4s were cultured in complete RPMI-1640 medium (Life Technologies, Grand Island, NY, USA) supplemented with 10% fetal calf serum (FCS) and antibiotics, penicillin, and streptomycin. Other human tumor cell lines, L428 (Hodgkin’s lymphoma), Daudi (Burkitt lymphoma), HeLaS3 (cervical epithelioid carcinoma), SAS (tongue squamous carcinoma) (25), SK-BR3 (breast adenocarcinoma) (26) and RMG1 (ovarian clear cell carcinoma) (27) were also provided by Keio University. L428, Daudi, HeLaS3, and SAS cells were cultured in complete RPMI-1640 medium with 10% FCS and antibiotics, and SK-BR3 cells were cultured in complete Dulbecco’s Modified Eagle’s Medium with 10% FCS and antibiotics.

### *In Vivo* Human Tumor Transplantation Model

HSC4 cells (1.5 × 10^6^, 100 µL PBS) were inoculated subcutaneously into HSC-NOG hIL-6 Tg mice or HSC-NOG non-Tg mice at 12–14 weeks after HSC transplantation. Solid tumor size was measured twice a week using a caliper and calculated using the following formula: tumor volume (mm^3^) = 1/2 × length (mm) × [width (mm)]^2^. Human MNCs in the tumor, spleen, and peripheral blood (PB) in the mice were analyzed 30–51 days posttumor inoculation when the tumor volume reached 2000 mm^3^.

### Preparation of Human Immune Cells from Human Tumors Engrafted in HSC-NOG Mice

Peripheral blood was collected from the abdominal vein of HSC-NOG-hIL-6 Tg or HSC-NOG non-Tg mice under anesthesia at the time of sacrifice. Blood plasma was separated by centrifugation. BM cells were obtained by flushing femurs with 3 mL PBS with 0.1% bovine serum albumin (BSA). Splenic cells were prepared by crushing the tissues between two frosted slides, and the tissue debris was removed using a 100-µm nylon mesh. Solid tumors were dissociated using a gentleMACS™ dissociator (Miltenyi Biotec, Bergisch Gladbach, Germany) in RPMI-1640 medium with collagenase IV (1 mg/mL; Sigma-Aldrich, St. Louis, MO, USA) and DNase I (0.1 mg/mL; Sigma-Aldrich), and subsequently incubated for 30 min at 37°C under gentle rotation. These steps were repeated twice. After dissociation, cells were filtered through a 70-µm mesh filter (BD Bioscience, Franklin Lakes, NJ, USA) to remove the debris. Mouse red blood cells (RBCs) were eliminated with RBC lysis buffer (stock solution contained 155 mM NH_4_Cl, 10 mM KHCO_3_ and 0.1 mM EDTA. This solution was diluted 4:1 with Dulbecco’s PBS before use). After washing, cell pellets were resuspended with PBS containing 0.1% BSA.

### Flow Cytometry

Cell viability was assessed by Trypan blue exclusion. Numbers of total leukocytes in PB were counted using a blood analyzer, Sysmex XT-2000i (Sysmex, Kobe, Japan), and the total blood volume was calculated from the body weight, assuming that mice contain 72 mL blood per kg body weight (28). The number of leukocytes in the total BM was calculated as 16 × the number of leukocytes in one femur (28). Single MNC suspensions were stained with the appropriate antibodies for 20 min at 4°C in the dark. After washing with fluorescence-activated cell sorting (FACS) buffer (PBS, 0.1% BSA, 0.1% NaN_3_), the cells were resusupended in FACS buffer containing propidium iodide (1 µg/mL; Dojindo Molecular Technologies, Inc., Kumamoto, Japan) to exclude dead cells. We used a BD FACSCanto™ (BD Biosciences) and a BD FACSAriaII™ (BD Biosciences) for multicolor flow cytometric analysis with FACSDiva™ software (BD Biosciences); the data were analyzed using the FlowJo^®^ software program (ver. 7.6.1; Tree Star, Inc., Ashland, OR, USA). The following antibodies were used: anti-human CD33 -phycoerythrin (PE)-Cy7(WM-53) was purchased from eBioscience (San Diego, CA, USA); anti-human CD11b-FITC (ICRF44), anti-human CD14-FITC (HCD14), anti-human CD33-FITC (HIM3–4), anti-human CD66b-FITC (G10F5), anti-mouse CD45-FITC (30-F11), anti-HLA-DR-Alexa Flour-488 (L243), anti-human CD19-PE (G077F6), anti-human CD66b-PE (G10F5), anti-human CD124-PE (IL-4Rα), anti-human CD163-PE (GHI/61), anti-human CD335 (NKp46)-PE (9E2), anti-mouse CD45-PerCP-Cy5.5 (30-F11), anti-human CD3-PE-Cy7 (UCHT1), anti-human CD14-PE-Cy7 (HCD14), anti-human CD56-PE-Cy7 (HCD56), anti-human CD68-PE-Cy7 (Y1/82A), anti-human CD16 –allophycocyanin (APC) (3G8), anti-human CD56-APC (HCD56), anti-HLA-DR-APC (L243), anti-human CD14-APC-Cy7 (HCD14), anti-human CD45-APC-Cy7 (HI30), anti-mouse CD45-APC-Cy7 (30-F11), anti-human CD3-Brilliant Violet 421 (UCHT1), anti-human CD11b-Brilliant Violet 421 (ICRF44), anti-human CD163-Brilliant Violet 421 (GHI/61), and anti-human CD45-Brilliant Violet 510 (HI30) were purchased from BioLegend (San Diego, CA, USA).

### Intracellular Staining

To investigate the expression of Arg-1 in human monocytes and macrophages, human MNCs from tumor, spleen, and PB of HSC-NOG-hIL-6 Tg mice or HSC-NOG non-Tg mice were fixed in 2% formaldehyde (Nacalai Tesque, Kyoto, Japan) for 15 min at room temperature, and subsequently stained with anti-human CD68-PE-Cy7 (Y1/82A) and anti-human Arg-1-APC (Clone # 658922; R&D Systems) or APC-conjugated mouse IgG2b isotype control (MPC-11; BioLegend) in the presence of 0.5% saponin (Nacalai Tesque) for permeabilization. Stained samples were analyzed using a BD FACSAriaII™ flow cytometer (BD Biosciences).

### Histology and Immunohistochemistry

For immunohistochemical studies, the tumor, spleen, liver, BM, lung, skin, gut, and kidney of HSC-NOG-hIL-6 Tg mice or HSC-NOG non-Tg mice were fixed in Mildform 10MN formaldehyde solution (Wako Pure Chemical, Osaka, Japan) and embedded in paraffin. The samples were serially sectioned into 3-µm thicknesses using a microtome, and placed on silane-coated slides (Muto Pure Chemicals, Tokyo, Japan). Immunostaining was performed using a Leica Bond-Max automatic immunostainer (Leica Biosystems, Mount Waverley, VIC, Australia). Paraffin sections were dewaxed in a Bond Dewax solution and rehydrated in alcohol and Bond Wash solution (Leica Biosystems). Antigen retrieval was performed using a retrieval solution (ER1, 10 mM citrate buffer, pH 6), followed by endogenous peroxidase blocking. Detection was performed using a Bond Polymer Refine Detection system. Then, the sections were counterstained with hematoxylin. We used monoclonal anti-human CD68 (clone: PG-M1, DakoCytomation, Glostrup, Denmark) and monoclonal anti-human CD163 (clone: 10D6, Leica Biosystems Newcastle Ltd., Newcastle, UK) antibodies for immunohistochemical analyses (National Institutes of Health, Bethesda, MD, USA).

### Detection of mRNA

Total RNA was isolated using Isogen reagent (Nippon Gene, Tokyo, Japan). The first strand cDNA was synthesized using oligo (dT) primers and the Superscript III First-Strand Synthesis System (Thermo Fisher Scientific, Waltham, MA, USA). For semi-quantitive polymerase chain reaction (PCR) analysis, the following primers were used: *gapdh*, 5′-TTAAAAGCAGCCCTGGTGAC-3′ (sense) and 5′-CTCTGCTCCTCCTGTTCGAC-3′ (antisense) (29); *il-10*, 5′-GGGTTGCCAAGCCTTGTCTG-3′ and 5′-CGCCGTAGCCTCAGCCTG-3′ (30); *vegf*, 5′-CACACAGGATGGCTTGAAGA-3′ and 5′-AGGGCAGAATCATCACGAAG-3′ (29). Each mRNA was amplified with EX-Taq (Takara Bio, Inc., Kusatsu, Japan). The intensity of each band was measured using ImageJ software.

### *In Vitro* Carboxyfluorescein Succinimidyl Ester (CFSE) Proliferation Assay

For CFSE prolifration assays, human CD3^+^ T cells were purified from the spleen of HSC-NOG non-Tg mice at 16–24 weeks after HSC transplantation by MACS (Miltenyi Biotec). Human CD11b^+^ cells were sorted from tumor or spleen of HSC-NOG-hIL-6 Tg mice or NOG non-Tg mice using a BD FACSAriaII™ (BD Biosciences). CFSE proliferation analyses were performed using a CellTrace™ CFSE Cell Proliferation Kit (Thermo Fisher Scientific) according to the manufacturer’s instructions. Human CD3^+^ T cells were sorted from tumor-free HSC-NOG non-Tg mice, which were transplanted with HSCs from a different donor from those of CD11b^+^ human myeloid cells. Briefly, human CD3^+^ T cells were labeled with 1 µM CellTrace™ CFSE in PBS for 5 min at 37°C and washed three times with 0.1% BSA/PBS to remove excess CFSE. CFSE-labeled human CD3^+^ T cells (1 × 10^5^) were stimulated with immobilized anti-hCD3 (OKT3, 20 µg/mL) and anti-hCD28 (CD28.2, 2 μg/mL)antibodies (BioLegend) at 37°C in the presence or absence of 5 × 10^4^ human CD11b^+^ cells. On day 7, dilutions of CFSE in human CD3^+^ T cells were analyzed.

### BD™ Cytometric Beads Array (CBA)

To measure the human cytokines produced by human tumor cell lines, we used a CBA kit (BD Biosciences). Standards (2,500–10 pg/mL and blank) and 50 µL culture supernatant from each cell line were added to 50 µL capture beads and incubated for 1 h at room temperature in the dark. After incubation, 50 µL PE detection reagents were added and incubated for 2 h at room temperature in the dark. After washing, test samples and standards were resuspended in 300 µL wash buffer and analyzed using a BD FACSCanto™ flow cytometer (BD Biosciences). The data were analyzed using FCAP Array™ v3.0 software (BD Biosciences).

## Results

### Enhancement of Human Monocyte/Macrophage Development in HSC-NOG-hIL-6 Tg Mice

**Figure 1:**
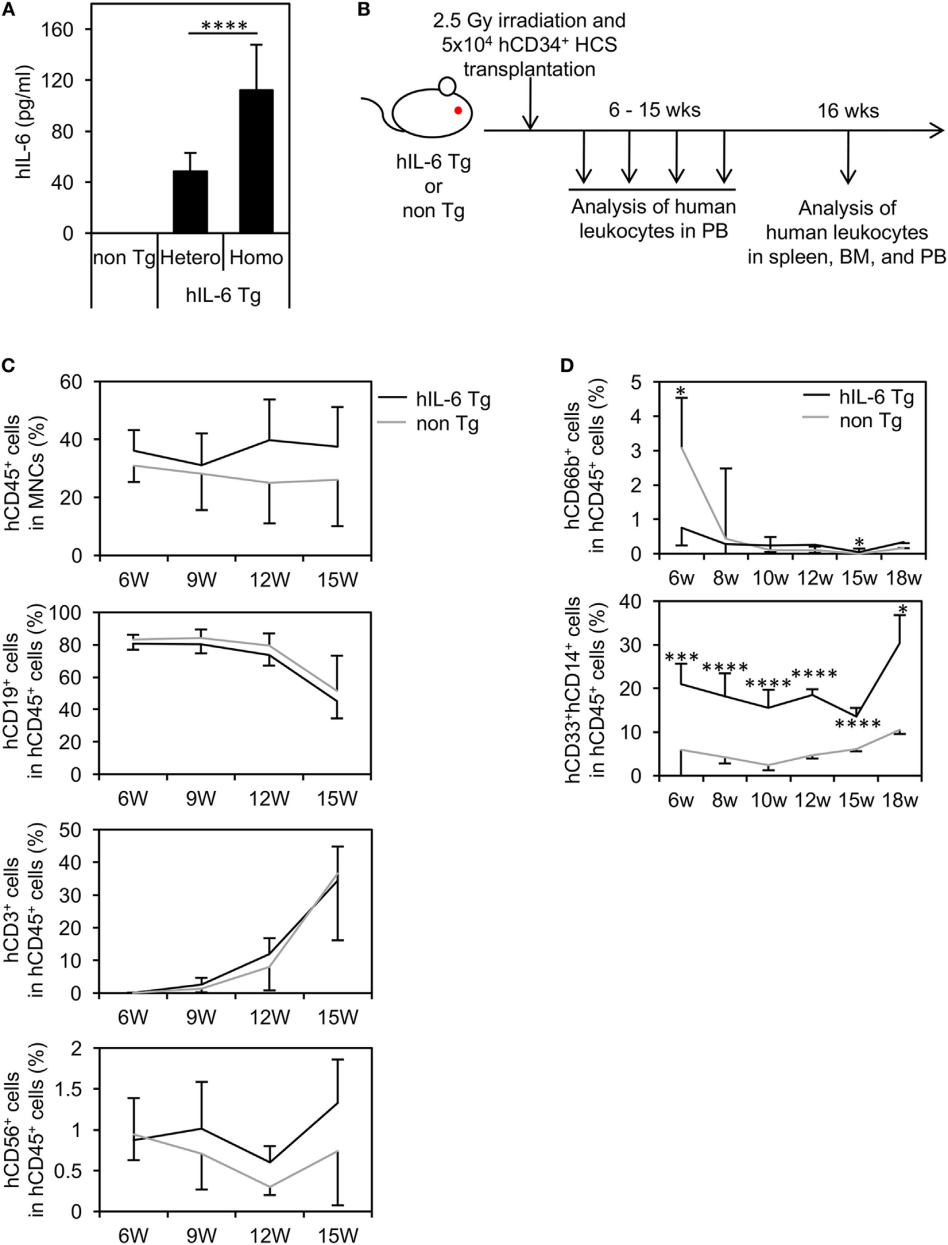
Human cell hematopoiesis in hematopoietic stem cell (HSC)-transplanted NOD/Shi-scid-IL-2Rγ*^null^* (NOG) substrain mice expressing transgenic (Tg) human interleukin 6 (hIL-6) (HSC-NOG-hIL-6 Tg). **(A)** Level of hIL-6. The levels of hIL-6 in the serum of NOG-hIL-6 homozygous Tg, heterozygous Tg, or NOG non-Tg mice were measured using enzyme-linked immunosorbent assay. The means ± SDs are shown (*n* = 25). Asterisks indicate statistical significance (****p < 0.0001). **(B)** NOG-hIL-6 Tg mice or NOG non-Tg mice were transplanted with 5 × 10^4^ cord blood human CD34^+^ HSCs 1 day after irradiation (2.5 Gy). Human leukocytes in peripheral blood (PB) were analyzed at the indicated time points. **(C)** The PB of HSC-NOG-hIL-6 Tg mice (black line, *n* = 6) and HSC-NOG non-Tg mice (gray line, *n* = 4) were analyzed by fluorescence-activated cell sorting (FACS) at 6–15 weeks after HSC transplantation. The frequencies of engrafted hCD45^+^ cells in all of the leukocytes and frequencies of each hematopoietic lineage in the hCD45^+^ cell population are shown. **(D)** The PB of HSC-NOG-hIL-6 Tg mice (black line, *n* = 5) and HSC-NOG non-Tg mice (gray line, *n* = 5) were FACS analyzed at 6–18 weeks after HSC transplantation. The frequencies of CD33^+^CD14^+^ monocytes/macrophages or hCD66b^+^ granulocytes in the hCD45^+^ cell population are shown (****p < 0.0001, *p < 0.05). The figures show representative data from three independent experiments. Student’s *t*-test was performed to analyze statistical significance.

The establishment of NOG-hIL-6 Tg mice was confirmed by measuring the production of hIL-6. Quantification by ELISA demonstrated that significant amounts of hIL-6 protein were present in plasma from both homozygous and heterozygous NOG-hIL-6 Tg, but not in NOG non-Tg mice. Homozygous NOG-hIL-6 Tg mice showed significantly higher IL-6 expression levels than heterozygous NOG-hIL-6 Tg mice (Figure 1A).

To investigate hematopoiesis of human cells in NOG-hIL-6 Tg mice, human CD34^+^ HSCs from umbilical CB were transferred into irradiated NOG-hIL-6 Tg mice and NOG non-Tg mice. The mice were bled for FACS analysis every 3 weeks from 6 weeks until 15 weeks after HSC transplantation (Figure 1B). The frequency of hCD45^+^ leukocytes in the total leukocyte population was slightly higher in HSC-NOG-hIL-6 Tg mice than in HSC-NOG non-Tg mice, but this difference did not reach significance (Figure 1C). There were no significant differences in human CD3^+^ T cells or CD19^+^ B cells (Figure 1C). The frequency of CD3^−^CD56^+^ NK cells increased in HSC-NOG-hIL-6 Tg mice at 8–9 weeks after HSC transplantation compared to that in HSC-NOG non-Tg mice; however, this increase did not reach statistical significance. This human CD45^+^CD3^−^NKp46^+^CD56^+^ NK cells in HSC-NOG-hIL-6 Tg mice consisted of three populations, CD56^dim^CD16^bright^ cytotoxic NK cells, cytokine producing CD56^+^CD16^−^ NK cells, and CD56^+^CD16^+^ NK cells. There were no significant differences between HSC-NOG-hIL-6 Tg mice and HSC-NOG non-Tg mice in frequencies and cellularties in those CD56^dim^CD16^bright^ and CD56^+^CD16^−^ NK cell fractions (Figure S1 in Supplementary Material) (31). Myeloid cells are generally classified into monocytes/macrophages and granulocytes. In the CD33^+^ myeloid cell fraction in the PB of HSC-NOG-hIL-6 Tg mice, CD33^+^CD14^+^ monocytes were markedly increased in HSC-NOG-hIL-6 Tg mice compared to NOG non-Tg mice, whereas CD66b^+^ granulocytes were barely detected in HSC-NOG-hIL-6 Tg mice, in a manner similar to HSC-NOG non-Tg mice (Figure 1D).

**Figure 2:**
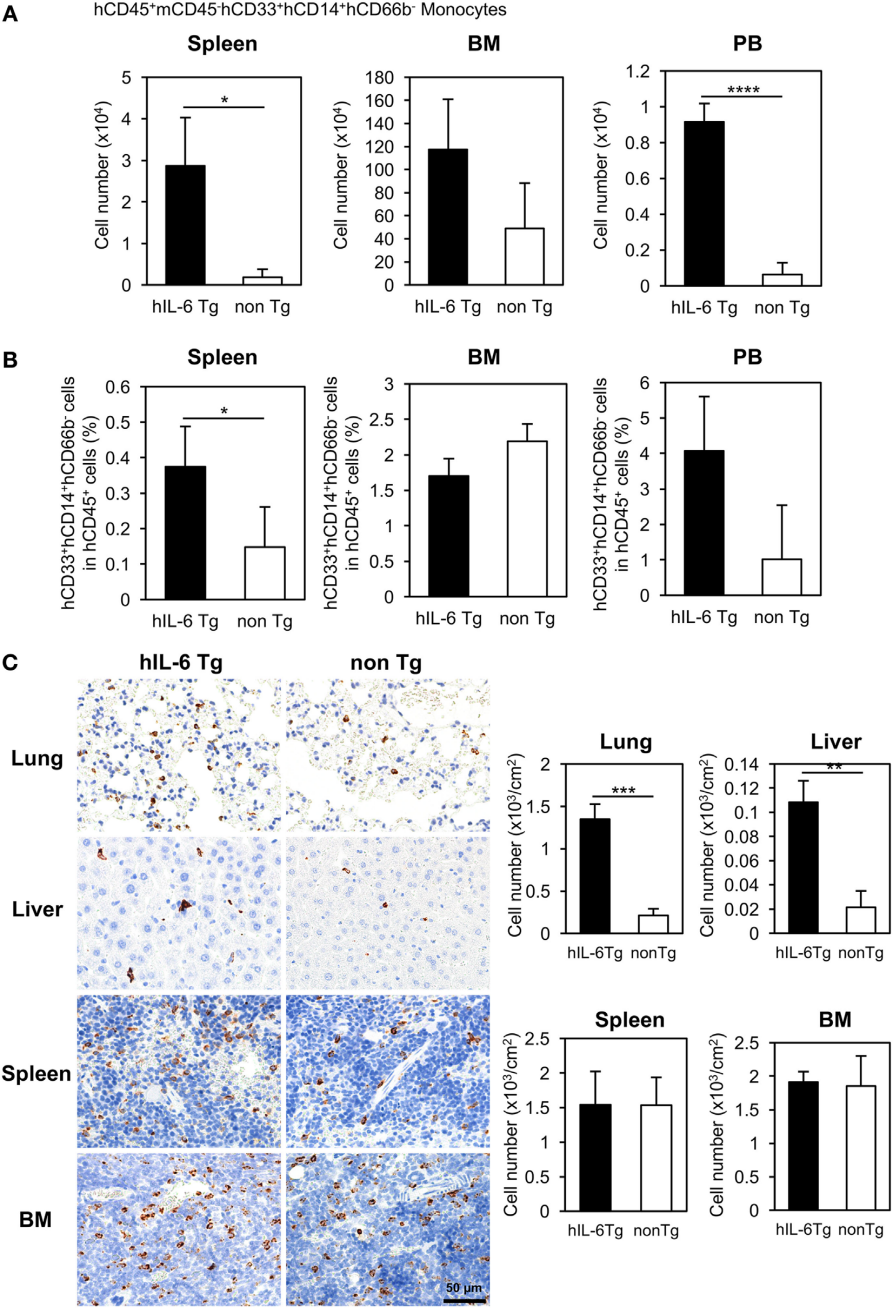
Development of human monocytes/macrophages in HSC-NOG-hIL-6 Tg mice. Mononuclear cells (MNCs) were prepared from the spleen, bone marrow (BM), and peripheral blood in HSC-NOG-hIL-6 Tg mice (filled column) and HSC-NOG non-Tg mice (open column) at 14 weeks after HSC transfer. The frequency of CD33^+^CD14^+^ monocytes/macrophages in hCD45^+^ leukocytes was obtained by flow cytometry **(A)**. The absolute number of CD33^+^CD14^+^ monocytes/macrophages was calculated by multiplying the number of total MNCs by the frequencies of each human subpopulation determined by fluorescence-activated cell sorting (FACS) **(B)**. The means ± SDs are shown (*n* = 3). Student’s *t*-test was performed to analyze the statistical significance. Asterisks indicate the statistical significance (*p < 0.05, ****p < 0.0001). **(C)** The distributions of human macrophages in HSC-NOG-hIL-6 Tg mice were assessed in paraffin-embedded lung, liver, spleen, and BM of HSC-NOG-hIL-6 Tg mice and HSC-NOG non-Tg mice; the tissues were sliced, mounted on slides, and stained with peroxidase-conjugated anti-human CD68 antibody. For enumeration of CD68^+^ macrophages in lung, liver, spleen, and BM, the number of signals in three different view fields in a representative tissue section was counted and divided by the area of the section using a BZ-9000 microscope (Keyence, Tokyo, Japan). The average number per unit area (cm^2^) is shown. An asterisk indicates statistical significance based on Student’s *t*-test (**p < 0.01, ***p < 0.001).

Flow cytometric analysis revealed that ~five or three-fold increases in the numbers of total human CD45^+^ cells in the spleen or BM of HSC-NOG-hIL-6 Tg mice, respectively (Figure S2 in Supplementary Material). The increase of monocytes/macrophages from HSC-NOG-hIL-6 Tg mice was more profound in the spleen and PB than in the BM (Figure 2A). The total cell number of CD33^+^CD14^+^CD66b^−^ monocytes was ~15.4-fold (spleen, PB) or 2.4-fold (BM) higher in HSC-NOG-hIL-6 Tg mice than in HSC-NOG non-Tg mice (Figure 2B). These results suggest that development of human monocytes/macrophages was enhanced in NOG-hIL-6 Tg mice. To analyze the distribution of human macrophages in various tissues, tissue sections of lung, liver, spleen, and BM from HSC-NOG-hIL-6 Tg or HSC-NOG non-Tg mice were stained with peroxdase-conjugated antibody against human CD68 (Figure 2C). The densities of CD68^+^ macrophages were higher in lung and liver from HSC-NOG-hIL-6 Tg mice than in those from HSC-NOG non-Tg mice, whereas the cell density in the spleen and BM of HSC-NOG non-Tg mice were comparable with those in HSC-NOG-hIL-6 Tg mice (Figure 2C). There were few CD68^+^ cells in the gut, skin, and brain from HSC-NOG-hIL-6 Tg and NOG non-Tg mice (data not shown). Hence, the increase of the cellularity of human monocytes/macrophages in the spleen (Figure 2A) is due to the increase of the absolute number of total CD45^+^ human cells.

### Characterization of Differentiated Human Monocytes and Macrophages in NOG-hIL-6 Tg Mice

**Figure 3:**
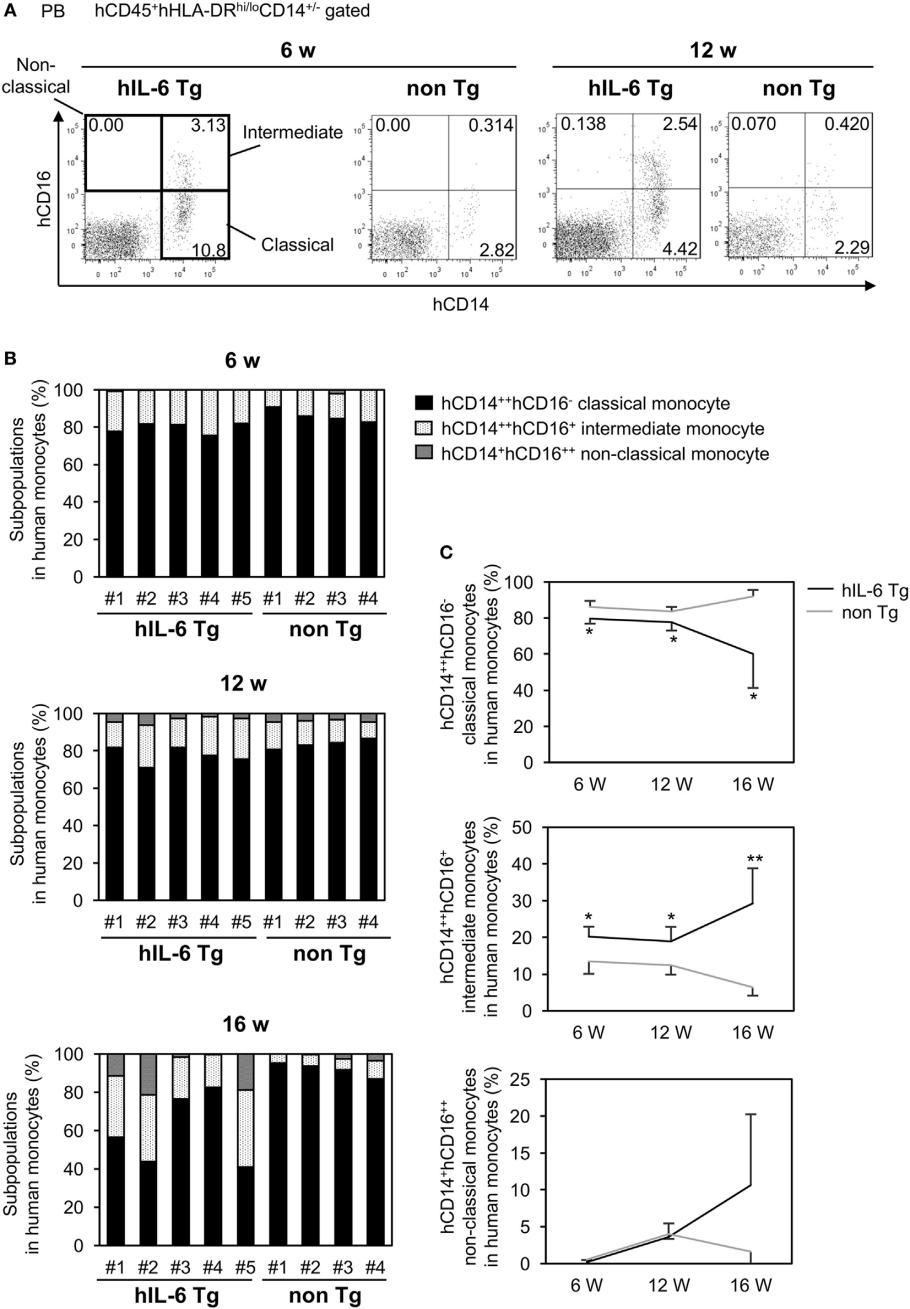
Subpopulations in peripheral blood (PB) monocytes in HSC-NOG mice. HSC-NOG-hIL-6 Tg (*n* = 6) and HSC-NOG non-Tg mice (*n* = 5) were bled at 6, 12, and 16 weeks after HSC transplantation, and numbers of mononuclear cells (MNCs) in PB were analyzed by FACS. **(A)** FACS plots of human monocyte subpopulations. Representative data from three independent experiments are shown. **(B)** The frequencies of subpopulations (CD14^++^CD16^−^ classical monocytes, CD14^++^CD16^+^ intermediate monocytes, and CD14^+^CD16^++^ non-classical monocytes) in the human monocytes of individual mice are shown. Human monocytes were defined as previously described (32, 34). **(C)** The kinetics of classical monocytes and intermediate monocytes in HSC-NOG-hIL-6 Tg mice (black line) and HSC-NOG mice (gray line). Student’s *t*-test was performed to assess statistical significance. Asterisks indicate statistical significance (**p < 0.01, *p < 0.05).

Based on expression of CD14 and CD16 (FcγRIII), human monocytes are classified into three subpopulations: CD14^++^CD16^−^ classical monocytes, CD14^+^CD16^++^ non-classical monocytes, and CD14^++^CD16^+^ intermediate monocytes (32, 33). In normal human PB, up to 90% of blood monocytes are CD14^++^CD16^−^ classical monocytes, and these cells can differentiate into CD14^+^CD16^++^ non-classical monocytes. We investigated the composition of human monocytes in PB from HSC-NOG-hIL-6 Tg and HSC-NOG non-Tg mice (Figure 3A). Nearly 80% of human monocytes were CD14^++^CD16^−^ classical monocytes at 6 weeks after HSC transplantation both in HSC-NOG-hIL-6 Tg and HSC-NOG non-Tg mice (Figure 3B). However, the populations decreased to 60% in HSC-NOG-hIL-6 Tg at 16 weeks after HSC transplantation in HSC-NOG-hIL-6 Tg (Figures 3B,C). The CD14^++^CD16^+^ intermediate monocytes increased and constituted about 30% in HSC-NOG-hIL-6 Tg mice at 16 weeks. In some individual mice in HSC-NOG-hIL-6 Tg mice, ~10% of monocytes were non-classical monocytes at 16 weeks. These monocyte populations were relatively stable in HSC-NOG non-Tg mice (Figures 3B,C).

**Figure 4:**
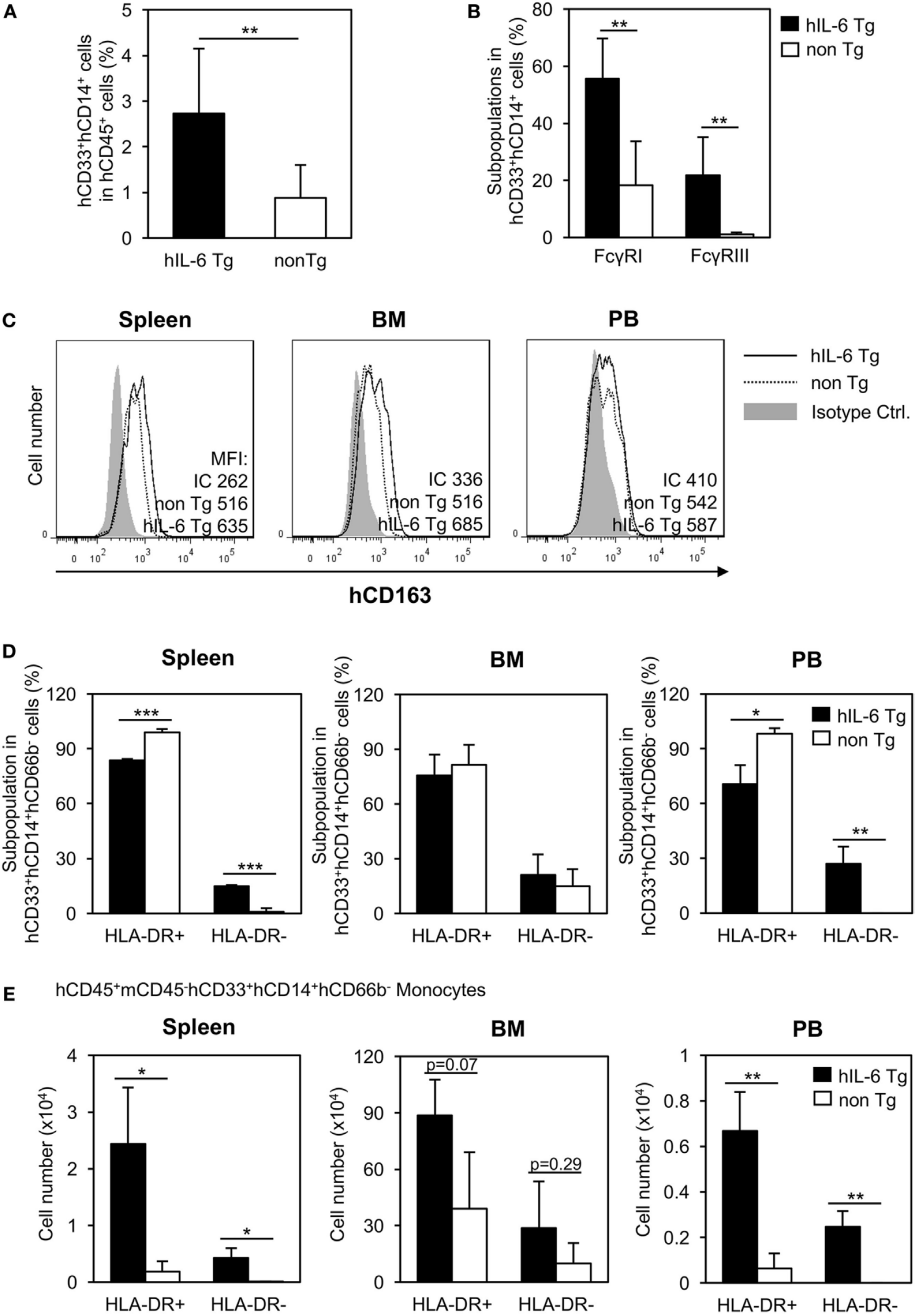
Characterization of human monocytes/macrophages in HSC-NOG-hIL-6 Tg mice. CD33^+^CD14^+^ cells in NOG-hIL-6 Tg mice (*n* = 6) or HSC-NOG non-Tg mice (*n* = 5) were FACS analyzed at 16 to 18 weeks after HSC transplantation. **(A)** The frequency of CD33^+^CD14^+^ monocytes/macrophages in the hCD45^+^ leukocytes is shown. An asterisk indicates the statistical significance according to the Student’s *t*-test (**p < 0.01). **(B)** Expression of Fcγ receptors on human monocytes/macrophages. Frequencies of hFcγRI- and hFcγRIII-expressing cells in splenic CD33^+^CD14^+^ monocytes/macrophages are shown (**p < 0.01). **(C)** Expression of hCD163 in human monocytes/macrophages. CD33^+^CD14^+^ cells in spleen, bone marrow (BM), and peripheral blood (PB) from HSC-NOG-hIL-6 Tg mice or HSC-NOG non-Tg mice were FACS analyzed for CD163 expression at 20 weeks after HSC transfer. (Bold line: HSC-NOG-hIL-6 Tg, broken line: HSC-NOG non-Tg). **(D,E)** Development of HLA-DR^+^ and HLA-DR^−^ human monocytes/macrophages in HSC-NOG-hIL-6 Tg mice. HLA-DR expression in CD33^+^CD14^+^ cells in spleen, BM, and PB in HSC-NOG-hIL-6 Tg mice (*n* = 3) or HSC-NOG non-Tg mice (*n* = 3) was analyzed at 14 weeks after HSC transplantation. The frequencies **(D)** and the absolute cell number **(E)** are shown (*p < 0.05, **p < 0.01, ***p < 0.001).

Next, we analyzed the spleen of HSC-NOG-hIL-6 Tg mice and confirmed that development of human CD33^+^CD14^+^ monocytes was promoted in the spleen (Figure 4A). Next, we investigated expression of several molecules, including the Fc receptors. Expression of Fc receptors represents the functionality of monocytes/macrophages to mediate phagocytosis. In HSC-NOG non-Tg mice, the frequencies of CD33^+^CD14^+^ monocytes/macrophages expressing FcγRI and FcγRIII were about 20 and 0%, respectively, in the total population of CD33^+^CD14^+^ human cells. By contrast, 60 and 20% of human monocytes/macrophages expressed FcγRI and FcγRIII, respectively, in HSC-NOG-hIL-6 Tg mice (Figure 4B). Macrophages consist of heterogeneous functional subpopulations, which are characterized by different cytokine production patterns and different expression profiles of various surface molecules. Nevertheless, they are roughly classified into inflammatory (classically activated) M1 macrophages and immunosuppresive (alternatively activated) M2 macrophages (35). As IL-6 is an essential factor for the generation of M2 macrophages (36, 37), we investigated whether the NOG-hIL-6 Tg or NOG non-Tg mice could develop differentiated monocytes/macrophages that resemble M2 macrophages. CD163 is a scavenger receptor that is thought to be a specific marker for delineating immunoregulatory M2 macrophages and immunosuppressive MDSCs and TAMs (38–41). We detected no significant differences in levels of CD163 in monocytes/macrophages from spleen, BM, or PB from HSC-NOG-hIL-6 Tg and NOG non-Tg mice (Figure 4C). A different M2 macrophage marker, IL-4Rα, was not expressed in human monocytes/macrophages in any of the tissues from either HSC-NOG-hIL-6 Tg or HSC-NOG non-Tg mice (Figure S3 in Supplementary Material). Next, we examined expression levels of HLA-DR (a class II HLA molecule), as HLA-DR expression is low or lost in immature human monocytes/macrophages such as MDSCs and TAMs. The frequencies (Figure 4D) and absolute cell numbers (Figure 4E) of HLA-DR-expressing or non-expressing monocytes/macrophages were compared in the spleen, BM, and PB from HSC-NOG-hIL-6 Tg and HSC-NOG non-Tg mice. Flow cytometoric analysis of spleen and PB demonstrated that HLA-DR^lo/−^ monocytes/macrophages constituted a significant subfraction of cells from the HSC-NOG-hIL-6 Tg mice, but not from the HSC-NOG non-Tg mice. In HSC-NOG-hIL-6 Tg mice, about 15.0 ± 0.46% or 26.9 ± 9.46% in CD33^+^CD14^+^CD66b^−^ human monocytes/macrophages were HLA-DR^lo/−^ cells in spleen or PB, respectively. Accordingly, the frequency of HLA-DR^+^ cells was lower in HSC-NOG-hIL-6 Tg mice than in HSC-NOG non-Tg mice (Figure 4D). Enumeration of HLA-DR^+^ and HLA-DR^lo/−^ human monocytes/macrophages showed significant increases in the numbers of HLA-DR^lo/−^ cells in the spleen and PB in HSC-NOG-hIL-6 Tg mice (Figure 4E). By contrast, the numbers of these cells in the HSC-NOG non-Tg mice were negligible. Both HLA-DR^+^ and HLA-DR^−^ populations were detected in the BM irrespective of the transgene. Neither the cell numbers nor frequencies of these fractions significantly differed between HSC-NOG-hIL-6 Tg and HSC-NOG non-Tg mice (Figures 4D,E). Collectively, these results imply that a portion of the human monocytes/macrophages that developed in NOG-hIL-6 Tg mice gained the unique HLA-DR^lo/−^ phenotype like MDSCs and TAMs.

### Generation of Human TAMs in Tumor-Engrafted HSC-NOG-hIL-6 Tg Mice

The detection of HLA-DR^lo/−^ myeloid cells in the spleen and PB in HSC-NOG-hIL-6 Tg mice prompted us to investigate the possibility that human immunosuppressive myeloid cells such as TAMs or MDSCs can be induced in NOG-hIL-6 Tg mice by the presence of transplanted human tumor cells. We analyzed the cytokine expression patterns in six different human tumor cell lines to identify the most suitable line for our experiments and found that HSC4, which is derived from human head and neck squamous cell carcinoma, exhibited high expression of IL-6, M-CSF, IL-8, and VEGF, and was the only line that produced IL-1β (Figure S4 in Supplementary Material).

**Figure 5:**
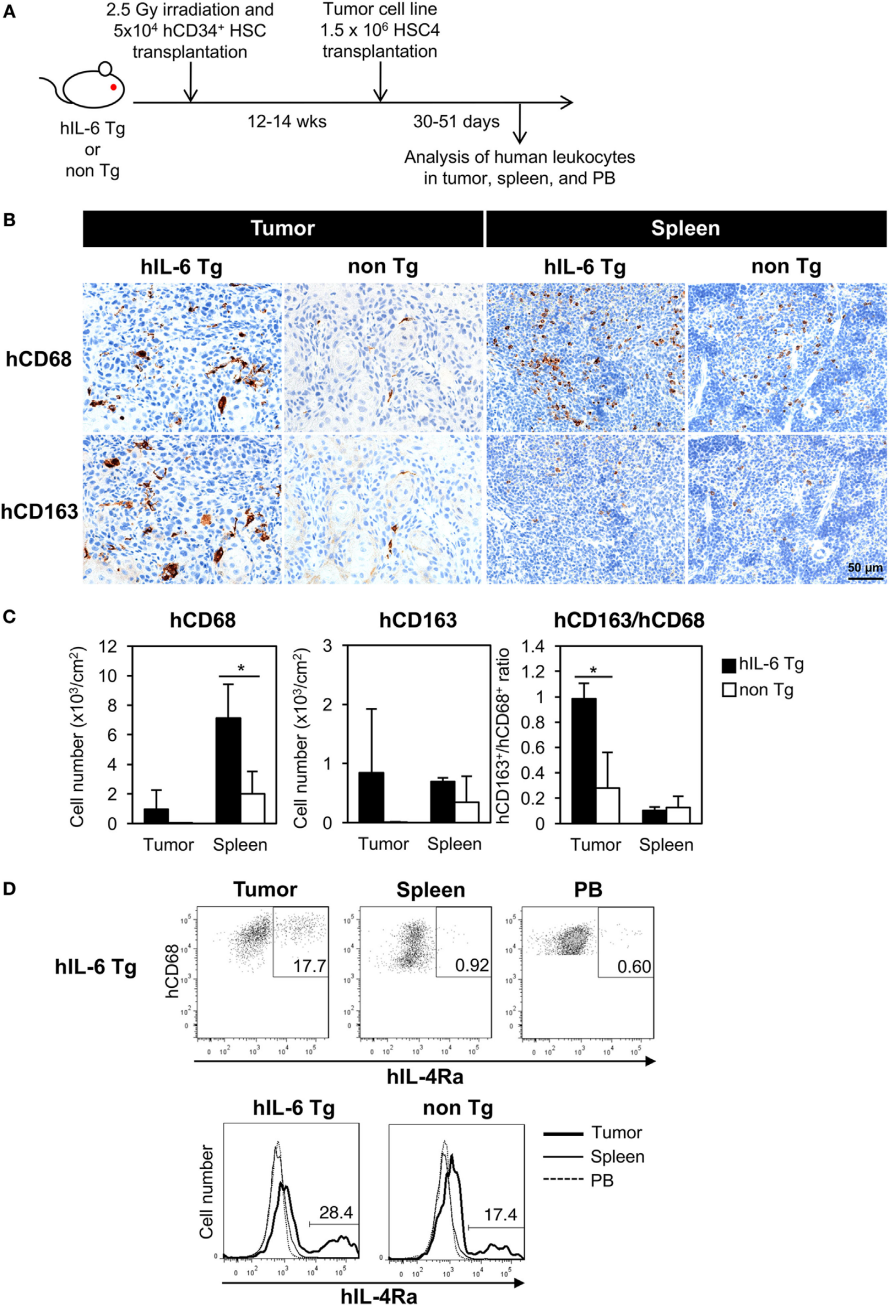
Development of human tumor-associated macrophages (TAMs) in tumor-engrafted HSC-NOG-hIL-6 Tg mice. **(A)** Schema of generation of tumor-bearing humanized mice. HSC-NOG-hIL-6 Tg or HSC-NOG non-Tg mice were inoculated with HSC4 (1.5 × 10^6^) at 12–14 weeks after HSC transplantation. Human leukocytes in tumor, spleen and peripheral blood (PB) were analyzed at 30–51 days after HSC4 engraftment. **(B)** Immunohistochemistry of human macrophages in tumor and spleen. Tumor-engrafted HSC-NOG-hIL-6 Tg or HSC-NOG non-Tg mice were analyzed at 36–51 days after tumor transplantation when the tumor size reached 2,000 mm^3^. Serial sections from tumor or spleen were stained with peroxidase-conjugated anti-CD68 or anti-CD163 antibodies. Each panel shows a representative image from three independent sections. **(C)** Enumeration of CD68^+^ or CD163^+^ macrophages. The number of signals in a whole tissue section was counted and divided by the area of the section using a BZ-9000 microscope (Keyence, Tokyo, Japan). Three independent sections were used, and the average number per unit area (cm^2^) is shown. The ratio of CD163^+^ to CD68^+^ cells was calculated using the average numbers. An asterisk indicates statistical significance based on Student’s *t*-test (*p < 0.05). **(D)** Expression of IL-4Rα in human macrophages. (Upper panels) FACS plots of IL-4Rα in hCD45^+^CD14^+^CD68^+^ macrophages in tumor, spleen, and PB of HSC4-engrafted HSC-NOG-hIL-6 Tg. (Bottom panel) Histogram of hIL-4Rα expression in hCD68^+^ macrophages in various tissues in HSC4-engrafted HSC-NOG-hIL-6 Tg and HSC-NOG non-Tg mice. Representative data from three experiments are shown.

HSC4 cells were subcutaneously implanted into HSC-NOG-hIL-6 Tg or HSC-NOG non-Tg mice at 12–14 weeks after CD34^+^ HSC transplantation, a time point when human monocytes, macrophages, T cells, B cells, and NK cells were detected in the PB. Infiltration of human macrophages into HSC4 tumor was analyzed at 30–51 days after HSC4 engraftment (Figure 5A). We conducted immunohistochemical analysis to detect human macrophages in HSC4-tumor using anti-CD68 or anti-CD163 antibodies, which are markers of macrophages or immunoregulatory macrophages, respectively. We detected a number of CD68^+^ macrophages in the spleen of both tumor-bearing HSC-NOG-hIL-6 Tg and HSC-NOG non-Tg mice, with greater numbers detected in HSC-NOG-hIL-6 Tg mice than in HSC-NOG non-Tg mice (Figures 5B,C). By contrast, analysis of tumor from the same mice demonstrated that a significant number of CD68^+^ macrophages infiltrated into the tumor in HSC-NOG-hIL-6 Tg mice, whereas few infiltrates were detected in HSC-NOG non-Tg mice (Figures 5B,C). Staining of serial sections with anti-CD163 antibody revealed that the majority of the tumor-infiltrating human macrophages in HSC-NOG-hIL-6 Tg mice were strongly positive for CD163 (Figures 5B,C). Indeed, enumeration of the positive signals in the sections revealed a much lower frequency of CD163-positive cells in human macrophages from HSC-NOG non-Tg mice (27.8 ± 0.28%) than in those from HSC-NOG-hIL-6 Tg mice (98.2 ± 0.12%) (Figure 5C). In the spleen, a large proportion of human macrophages had negative or weak CD163 expression (Figures 5B,C).

To characterize the human macrophages in HSC-NOG-hIL-6 Tg mice further, we examined the expression of IL-4Rα in human macrophages, as IL-4 is one of the factors supporting the differentiation of TAMs and expression of the IL-4 receptor is a marker for delineating TAMs in tumor (42, 43). Human macrophages in tumor, but not in the spleen or PB, expressed a significant amount of IL-4Rα (Figure 5D). This expression was detected in both HSC-NOG-hIL-6 Tg and HSC-NOG non-Tg mice.

The results imply that the human macrophages that developed in the NOG-hIL-6 Tg mice preferentially gained immunosuppressive phenotypes in the tumor.

### Functions of Immunosuppressive Myeloids in HSC-NOG-hIL-6 Tg Mice

**Figure 6:**
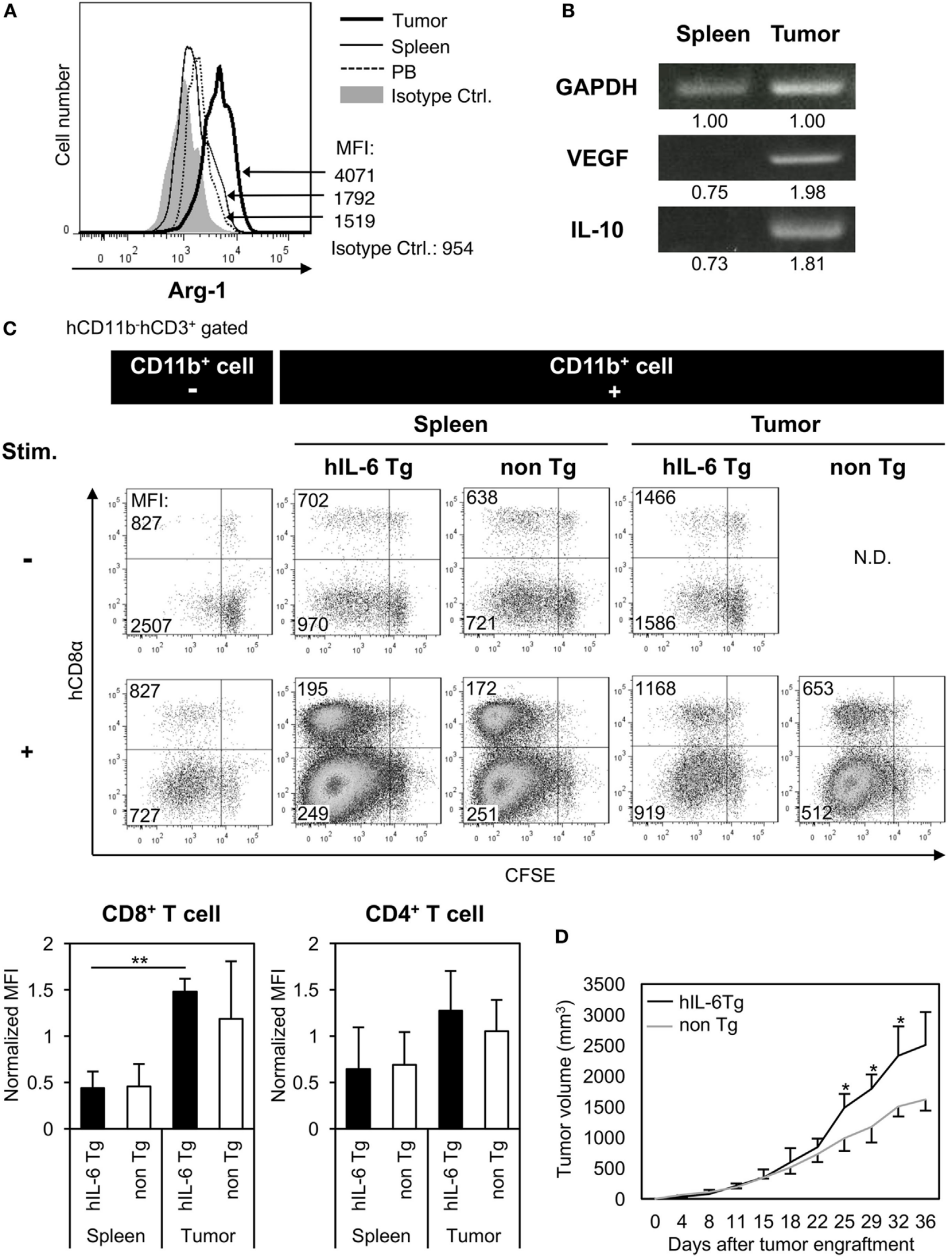
Immunosuppressive function of human tumor-associated macrophages (TAMs) in tumor-engrafted HSC-NOG-hIL-6 Tg mice. **(A)** Expression of human arginase-1 (Arg-1) in TAMs. Tumor-infiltrating cells and mononuclear cells (MNCs) from spleen and peripheral blood (PB), prepared from HSC4-engrafted HSC-NOG-hIL-6 Tg mice, were analyzed by intracellular staining with anti-hARG1 or isotype control. The expression of Arg-1 in CD14^+^CD68^+^ cells in tumor, spleen, and PB is shown. A representative fluorescence-activated cell sorting (FACS) plot from three independent experiments is shown. **(B)** Reverse transcription polymerase chain reaction (RT-PCR) for vascular endothelial growth factor (VEGF) and interleukin (IL)-10. hCD45^+^CD11b^+^ myeloid cells were purified from tumor and spleen in tumor-engrafted HSC-NOG-hIL-6 Tg or HSC-NOG non-Tg mice. After isolation of total RNA and synthesis of cDNA, VEGF and IL-10 were detected by PCR. The intensity of each band was measured using ImageJ software; normalized values are shown. Representative data from two independent experiments are shown. **(C)** Suppression of T cell activation by TAMs *in vitro*. hCD45^+^CD11b^+^ myeloid cells were sorted from tumor or spleen in tumor-engrafted HSC-NOG-hIL-6 Tg or HSC-NOG non-Tg mice. The cells were cultured with carboxyfluorescein succinimidyl ester (CFSE)-labeled CD3^+^ T cells from another non-tumor engrafted HSC-NOG non-Tg mouse in a 96-well plate followed by stimulation with or without immobilized anti-CD3 and anti-CD28 antibodies. T cell proliferation was analyzed by FACS on day 7 after staining with anti-CD3 and CD8. The number in each quadrant shows the mean fluorescence intensity (MFI) value. Representative data from three independent experiments are shown. To determine the degree of suppression, the MFI value of CD8^+^ T cells or CD4^+^ T cells, which at least proliferated one time, in the first quadrant (left top quadrant) or in the third quadrant (left bottom quadrant), respectively, was normalized by those of T cells in a control group, which were stimulated with anti-CD3/CD28 antibodies alone without CD11b^+^ cells. The mean ± SDs from three independent experiments are shown. Student’s *t*-test was performed to analyze the statistical significance (**p < 0.01). **(D)** Tumor growth in HSC-NOG-hIL-6 Tg or HSC-NOG non-Tg mice. HSC4 were transplanted into HSC-NOG-hIL-6 Tg mice (black, *n* = 3) or HSC-NOG non-Tg mice (gray, *n* = 3) at 14 weeks after HSC transplantation. Tumor growth was monitored over 36 days after tumor engraftment. The human CD34^+^ HSC cells came from different donor from Figure S3 in Supplementary Material. An asterisk indicates statistical significance based on Student’s *t*-test (*p < 0.05).

Host immune reactions against tumors, particularly T cell activation and proliferation, are suppressed by multiple mechanisms in patients with cancer. Several immunosuppressive factors, including Arg-1 or IL-10 produced from MDSCs and TAMs, are responsible for this suppression (20, 35). To investigate whether TAM-like cells differentiated in humanized mice have similar immunosuppressive functions to those in cancer patients, Arg-1 expression in CD14^+^CD68^+^ monocytes/macrophages was analyzed in the tumor, spleen, and PB of HSC-NOG-hIL-6 Tg mice or HSC-NOG non-Tg mice. Significant expression of human Arg-1 was detected in the tumor-infiltrating human monocytes/macrophages, whereas monocytes/macrophages in the spleen and PB showed modest expression (Figure 6A). Furthermore, reverse transcription polymerase chain reaction analysis showed hIL-10 or VEGF transcripts in human CD11b^+^ myeloid cells, which were sorted from the HSC4 tumor in HSC-NOG-hIL-6 Tg mice (Figure 6B). CD11b^+^ cells in the spleen from the same mice had no detectable amounts of IL-10 and VEGF transcripts (Figure 6B). Finally, to examine whether these human TAM-like cells have immunosuppressive function, the TAM-like cells were cultured with T cells *in vitro*, and the proliferation of T cells was assessed using CFSE assays. Total human CD11b^+^ myeloid cells were purified from the tumor or spleen of HSC-NOG-hIL-6 Tg mice or HSC-NOG non-Tg mice, as we could not obtain sufficient numbers of TAM-like cells when we gated HLA-DR^lo/−^ IL-4Rα^+^ CD163^+^ CD68^+^ cells. The cells were cultured with CFSE-labeled human T cells, which were purified from the spleen from different HSC-NOG non-Tg mice in the presence of immobilized anti-CD3/CD28 antibodies. The proliferation of T cells was analyzed by flow cytometry on day 7. Proliferation of CD8^+^ T cells was reduced in the groups that were cultured with human CD11b^+^ cells isolated from tumors of HSC-NOG-hIL-6 Tg mice. By contrast, CD11b^+^ cells from tumors of HSC-NOG non-Tg mice showed modest suppression. CD11b^+^ cells from spleen of HSC-NOG-hIL-6 Tg mice or HSC-NOG non-Tg mice had no suppressive activity on human T cells, rather enhanced proliferation (Figure 6C).

To demonstrate their suppressive function *in vivo*, tumor progression was compared between HSC-NOG-hIL-6 Tg and HSC-NOG non-Tg mice using HSC4 cells. The tumor size was measured from days 0 to 36 after tumor engraftment. Tumor progression was enhanced in HSC-NOG-hIL-6 Tg mice compared to that in HSC-NOG non-Tg mice (Figure 6D).

## Discussion

We generated a novel NOG substrain, which expresses hIL-6 in a constitutive manner. We demonstrated that this strain has several intriguing features compared to the parental NOG mice, particularly under tumor-engrafted pathological conditions.

Enhanced differentiation of human monocytes/macrophages in NOG-hIL-6 Tg mice after human HSC transplantation is in line with one major activity of the IL-6 cytokine. Previous *in vitro* studies have demonstrated that the addition of anti-IL-6 receptor (IL-6R) antibody inhibits monocytic colony formation; conversely, exogenous IL-6 stimulates monocytic colony formation together with GM-CSF (44). Thus, it is probable that IL-6 in NOG-hIL-6 Tg mice stimulates monocyte differentiation in the BM. However, considering that the increase in monocytes/macrophages was greater in the periphery than in the BM in HSC-NOG-hIL-6 Tg mice (Figure 2), it is more likely that exogenous IL-6 promoted accumulation of human monocytes/macrophages in the periphery in HSC-NOG-hIL-6 Tg mice. Indeed, a recent study demonstrated that IL-6 and M-CSF coordinatedly promote macrophage differentiation from monocytes by regulating the expression of M-CSF receptors in the monocytes (45). Exogenous IL-6 likely regulates development of monocytes/macrophages at both the BM and peripheral levels.

Recent studies have implied a role for IL-6 in the differentiation of M1/M2 macrophages. Mauer et al. reported that IL-6 induces IL-4Rα in mouse macrophages, augmenting the IL-4-induced polarization of M2 macophages (46). However, the absence of IL-4Rα expression and the modest expression of CD163 in human monocytes/macrophages in HSC-NOG-hIL-6 Tg mice suggests that they are not always differentiated into M2 macrophages. Other cytokines such as human M-CSF might be indispensable for the polarization toward M2 lineage in humanized mouse models. Indeed, the study of Mauer et al. used bone marrow-derived macrophages, which were induced by M-CSF *in vitro* for 8–10 days. Since mouse M-CSF does not cross-react efficiently on human macrophages (data not shown), the differentiation of human macrophages in HSC-NOG-hIL-6 Tg mice may be still incomplete and fail to express IL-4Rα in response to IL-6.

An interesting phenotype of human monocytes/macrophages in NOG-hIL-6 Tg mice is that they contain a significant number of HLA-DR^lo/−^ cells. Such HLA-DR^lo/−^ cells were detected in the BM of both HSC-NOG-hIL-6 Tg and HSC-NOG non-Tg mice, but not in the periphery in HSC-NOG non-Tg mice. This result suggests that HLA-DR^lo/−^ cells in the BM are normal immature monocytes/macrophages, whereas those in the periphery in HSC-NOG-hIL-6 Tg mice represent an unusual population. There would be two different mechanisms for the induction of HLA-DR^lo/−^ cells. One is that HLA-DR^lo/−^ cells in BM retained the phenotype even after migrating to the periphery. Another possibility is that HLA-DR^+^ cells lost the expression of HLA-DR. Absence of HLA-DR is considered a marker for defining immunosuppressive myeloid cells such as TAMs or MDSCs (35). Therefore, NOG-hIL-6 Tg mice provide a unique environment that allows the spontaneous development and maintenance of immunosuppressive myeloid cells or their precursor cells, unlike mice from other strains. We previously established NOG-hGM-CSF/IL-3 Tg mice, which showed enhanced human myelopoiesis including monocytes/macrophages (13). In this model, the HLA-DR^lo/−^ population was not evident (data not shown), which suggests the unique role of IL-6 in inducing HLA-DR^lo/−^ monocytes/macrophages.

Tumor engraftment experiments further demonstrated distinctions between NOG-hIL-6 Tg mice and NOG non-Tg mice. Few CD68^+^ macrophages infiltrated the tumor in HSC-NOG non-Tg mice, whereas an abundance of CD68^+^ macrophages infiltrated the tumor in HSC-NOG-hIL-6 Tg mice. This result was likely due to the poor development of human monocytes/macrophages in HSC-NOG non-Tg mice. Our immunohistochemical analysis demonstrated that almost all of the intratumoral macrophages expressed CD163 in HSC-NOG-hIL-6 Tg mice. The strong intensity of CD163 in intratumoral macrophages compared to splenic macrophages indicates that TAM-like cells with immunosuppressive activity differentiated in the tumors of HSC-NOG-hIL-6 Tg mice. As the increase in CD163 was intratumor specific, tumor-derived stimuli were necessary for inducing the differentiation into TAM-like cells. In contrast to the prevalence of CD163^+^ macrophages in tumor-bearing HSC-NOG-hIL-6 Tg mice, the frequency of CD163^+^ cells in intratumoral CD68^+^ cells was low in HSC-NOG non-Tg mice despite the small numbers of infiltrating human macrophages. This suggests that the local cytokine milieu in tumor alone is not always sufficient to induce differentiation of human macrophages into TAM-like cells. One explanation for this result is that the level of IL-6 in this tumor might be too low to induce TAM-like cells in HSC-NOG non-Tg mice. Another possibility is that systemic IL-6 in NOG-hIL-6 Tg mice predisposes human macrophages to differentiate into TAM-like cells upon exposure to stimuli from the tumor microenvironment. This notion is consistent with the accumulation of HLA-DR^lo/−^ cells in tumor-free HSC-NOG-hIL-6 Tg mice. Tumor inoculation and formation of microenvironment may further induce alternations of immunological characters in local human macrophages, resulting in the accumulation of CD163^hi^IL-4Rα^+^ TAM-like cells in an intratumor specific manner, but not in spleen or PB.

The increase in CD163 expression levels in intratumoral TAM-like cells in HSC-NOG-hIL-6 Tg mice implies that this model is relevant to the clinical course of cancer. Accumulating evidence has suggested that not only a high frequency of CD163^+^CD68^+^ TAMs, but also a high amount of CD163 in serum or in tumor are correlated with a poor prognosis in patients with cancer (47–49). Thus, the upregulation of CD163 in intratumoral TAM-like cells in NOG-hIL-6 Tg mice indicates that the tumor microenvironment in progressive cancer was recapitulated in this mouse model, at least in part. Importantly, induction of TAM-like cells was not HSC4-cell line specific. We have engrafted SAS and L428 with different cytokine profiles (Figure S4). An ovarian clear cell carcinoma cell line, RMG-I, was also tested. RMG-I and SAS formed tumor in NOG-hIL-6 Tg mice and flowcytometric analysis demonstrated the induction of CD163^+^IL-4Rα^+^TAM-like human myeloid cells as in HSC4-tumor (Figure S5 in Supplementary Material).

Myeloid-derived suppressor cells, another type of immunosuppressive myeloid cells, are distributed systemically in cancer patients, unlike TAMs, which localize in tumors. We examined whether MDSCs, defined as HLA-DR^lo/−^ cells in monocytes gated by CD33^+^CD11b^+^CD14^+^CD66b^−^ (35, 50), were expanded in tumor-bearing HSC-NOG-hIL-6 Tg mice; however, we failed to detect increased numbers of HLA-DR^lo/−^ cells in spleen and PB. In addition, CD163 expression was not elevated in tumor-bearing HSC-NOG-hIL-6 Tg mice (data not shown), and there were no transcripts of VEGF and IL-10 in splenic monocytes in HSC-NOG-hIL-6 Tg mice. Thus, induction of MDSC-like cells was not efficiently induced in HSC-NOG-hIL-6 Tg mice, although tumor-free HSC-NOG-hIL-6 Tg mice contained a significant number of HLA-DR^lo/−^ cells. Mounting evidence has suggested that many molecules are involved in the induction of MDSCs. Although IL-6 is one of the key molecules, exogenous supplementation of other cytokines such as GM-CSF and G-CSF, or pro-imflammatory molecules like IL-1β, S100A8, and S100A9, remains necessary for efficient induction and accumulation of human MDSCs (21–24, 51). Although HSC4 produces a part of these cytokines, the amounts may be insufficient.

The expression of Arg-1, IL-10, and VEGF in intratumoral macrophages in HSC-NOG-hIL-6 Tg mice suggests similarities with TAMs in cancer patients, and that they mediate immunosuppression in tumors. *In vitro* coculture with human T cells demonstrated that they have inhibitory activity against proliferation of human T cells, especially CD8^+^ T cells. In our experiments, we used total CD11b^+^ cells instead of purified TAMs, as the purification of TAMs did not yield a sufficient number for the assays. Nevertheless, the inhibition of T cell activation suggests that TAM-like cells with immunosuppressive functions can be induced in tumor in HSC-NOG-hIL-6 Tg mice, even if they are not completely identical to TAMs in cancer patients. Similarities between TAM-like cells in HSC-NOG-hIL-6 Tg mice and *bona fide* TAMs should be examined in the future by gene profiling or immunophenotyping.

Given the suppression of T cell activation *in vitro* by intratumoral macrophages in HSC-NOG-hIL-6 Tg mice, we suspect that they also have suppressive activity on human T cells in tumor to prevent anti-tumor immunity. Indeed, our tumor inoculation experiments showed that tumor growth was enhanced in HSC-NOG-hIL-6 Tg mice compared to that in HSC-NOG non-Tg mice. However, this result cannot be attributed solely to the generation of human TAM-like cells in HSC-NOG-hIL-6 Tg mice. First, the number of human TAMs was not sufficient, as mentioned above. Second, many mouse myeloid cells infiltrated into the human tumor. These cells contained murine TAMs and MDSCs to support tumor growth (data not shown). Thus, elimination of tumor-infiltraing mouse cells is indispensable. Furthermore, even without human HSC transplantation, HSC4 cells grew better in NOG-hIL-6 Tg mice than in NOG non-Tg mice (Figure S6 in Supplementary Material), which was most likely due to the direct effects of hIL-6 on HSC4 cells. Hence, the use of IL-6-independent tumor cells is necessary to clarify the immunosuppressive role of TAM-like cells *in vivo*. Although we screened several human cell lines without IL-6R and gp130, all of the cell lines were positive for their expression. Developing strategies to overcome these problems will facilitate reconstitution of the tumor microenvironment with minimum interference from mouse cells.

Immunosuppressive myeloid cells have been targets for cancer immune therapy. Our NOG-hIL-6 Tg mice partly enabled reconstitution of the tumor microenvironment, which included human TAMs. Sophistication of humanized mouse technology by development of new strains with other human cytokines will recapitulate the human tumor microenvironment, not only with TAMs but also with MDSCs, in combination with NOG-hIL-6 Tg mice. These mice provide a promising tool for drug development.

## Ethics Statement

All of the animal experiments were approved by the institutional animal care and use committee of the CIEA and were performed in accordance with guidelines set forth by the CIEA (11004, 14038R). All of the experiments using human cells were approved by the institutional ethical committee of the CIEA.

## Author Contributions

RI established a NOG-hIL-6 Tg mouse strain. AH conducted the experiments and analysis. IK helped with the HSC transfer. KK performed the histological analysis. MG operated the embryo manipulation. YK provided the human tumor cell lines and supervised the project. HS, MI, TT, and AH designed the experiments and completed the manuscript.

## Conflict of Interest Statement

The authors declare that the research was conducted in the absence of any commercial or financial relationships that could be construed as a potential conflict of interest.

## Abbreviations

IL-6, interleukin-6; NOG, NOD/ShiJic/scid/IL-2Rγ*^null^* mice; NOG-hIL-6 Tg, NOG mouse substrain expressing transgenic human IL-6; non-Tg, nontransgenic; HSC, hematopoietic stem cell; CB, cord blood; HSC-NOG, human hematopoietic stem cell-transplanted NOG mouse; PB, peripheral blood; BM, bone marrow; MNC, mononuclear cell; RT-PCR, reverse transcription polymerase chain reaction.
