# Supplementary material for: Generation of Human Immunosuppressive Myeloid Cell Populations in Human Interleukin-6 Transgenic NOG Mice
Source: Front Immunol. 2018 Feb 2;9:152. doi: 10.3389/fimmu.2018.00152 (PMC5801301; doi:10.3389/fimmu.2018.00152)
Supplement: Supplementary file 1 [file Presentation_1.PDF]

Supplemental Figure 1

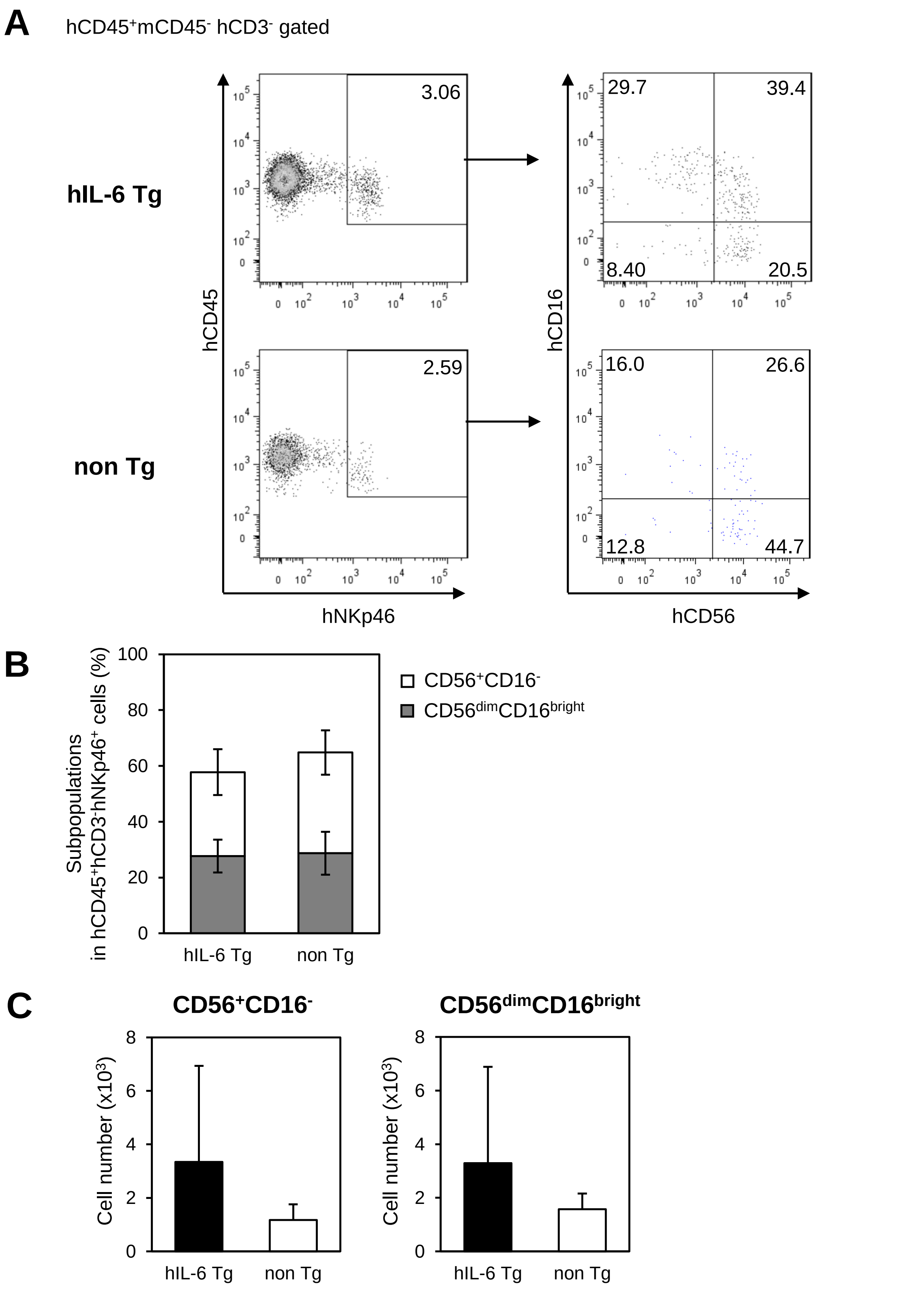

Supplemental Figure 1. Flow cytometric analysis of human natural killer (NK) cells in HSC-NOG-hIL-6 Tg mice. PB was stained with anti-human NKp46, CD16, and CD56 antibodies. **(A)** FACS plot of human NK cells. **(B)** Frequency of $CD56^{+}CD16^{-}$ and $CD56^{dim}CD16^{bright}$ cells in human $CD45^{+}CD3^{-}NKp46^{+}$ NK cells. (hIL-6 Tg: n=5, non Tg: n=5) **(C)** Cell numbers of $CD56^{+}CD16^{-}$ or $CD56^{dim}CD16^{bright}$ NK cells in human $CD45^{+}CD3^{-}$ cells. (hIL-6 Tg: n=5, non Tg: n=5)

Supplemental Figure 2

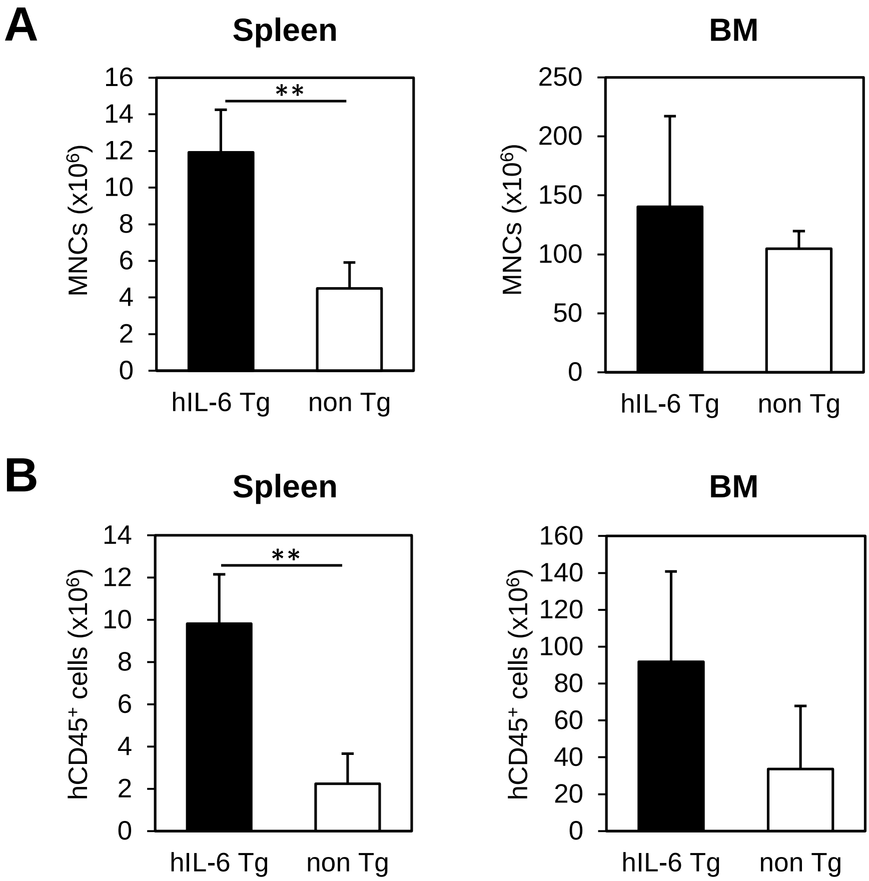

Supplemental Figure 2. The numbers of total mononuclear cells (MNCs) **(A)** and human $CD45^+$ cells **(B)** in HSC-NOG-hIL-6 Tg (n=3) or -non Tg (n=3) mice. The cells were isolated from spleen or BM of each mouse and analyzed by FACS. The graphs show the cell numbers in each tissue. (**p<0.01)

Supplemental Figure 3

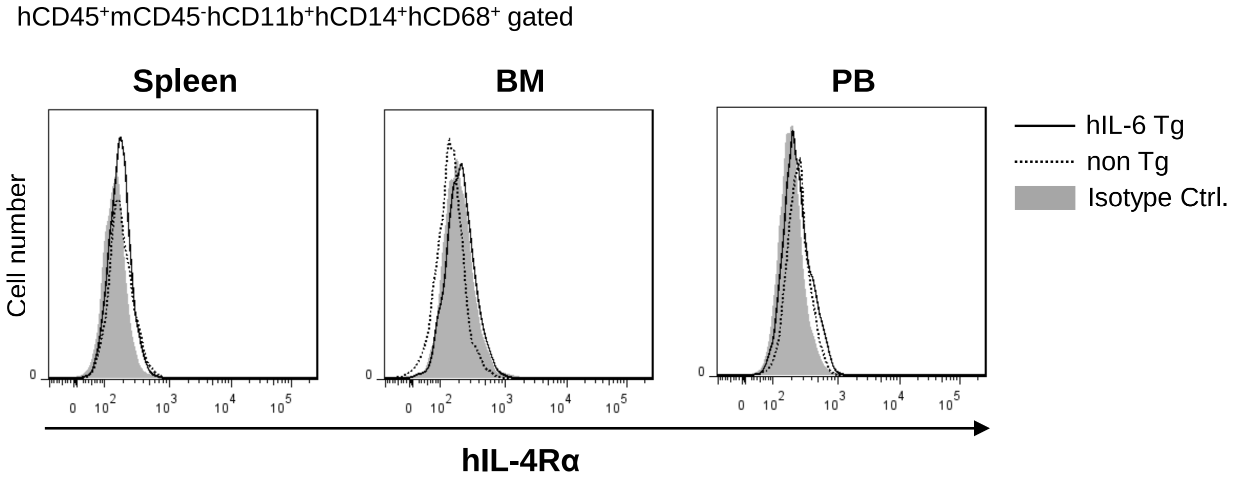

Supplemental Figure 3. Expression of hIL-4Rα in human macrophages in spleen, BM, and PB in HSC-NOG-hIL-6 Tg or -non-Tg mice.

Supplemental Figure 4

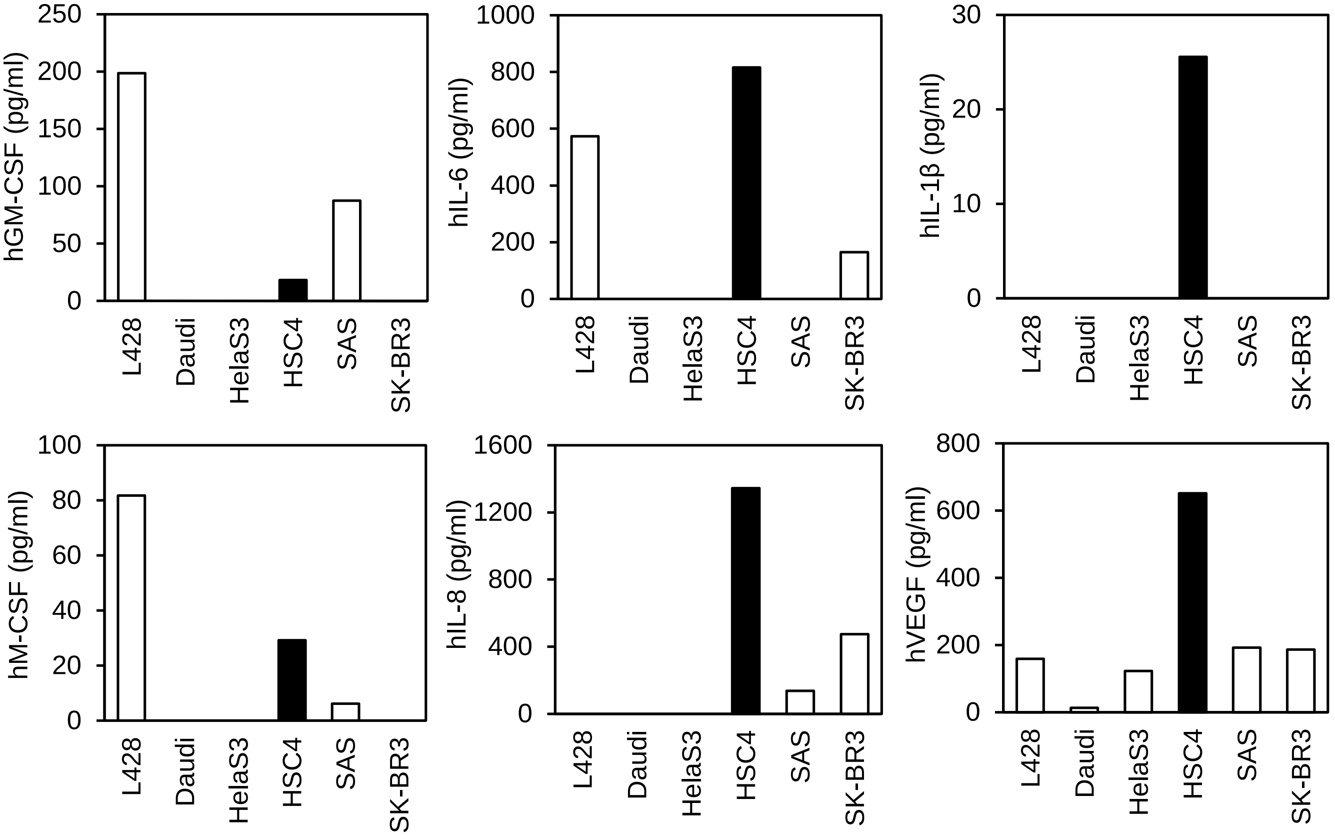

Supplemental Figure 4. Cytokine expression in human tumor cell lines. L428, Daudi, HelaS3, HSC4, SAS, and SK-BR3 were cultured for 24 h (2×10^5 in 500 µL). The amount of released cytokines was measured using a BD™ cytometric bead array (CBA). The graph shows representative data from three independent experiments.

Supplemental Figure 5

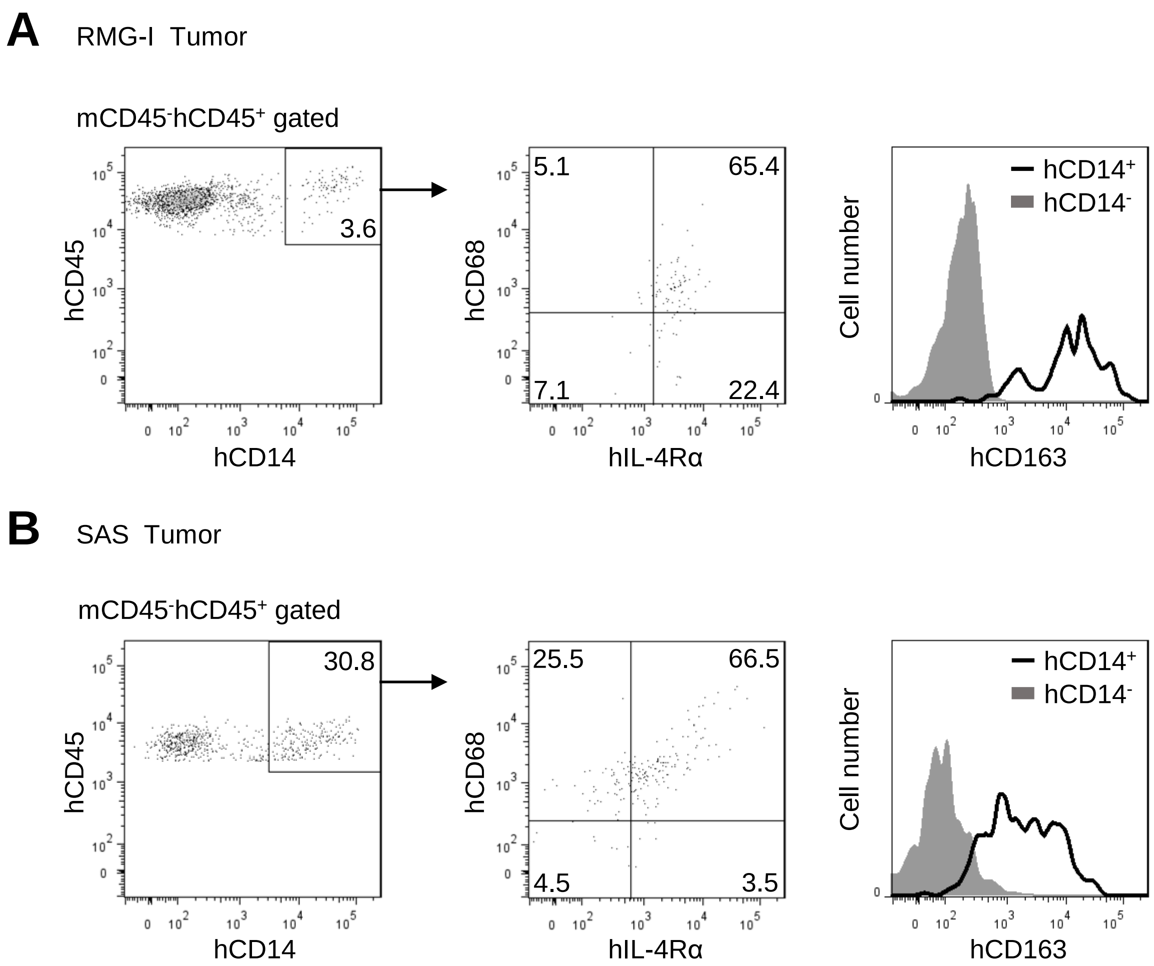

Supplemental Figure 5. Induction of human TAMs in HSC-NOG-hIL-6 Tg mice engrafted with human tumor cell lines RMG-I, derived from ovarian clear cell carcinoma **(A)** and SAS, derived from human tongue squamous cell carcinoma **(B)**.

Supplemental Figure 6

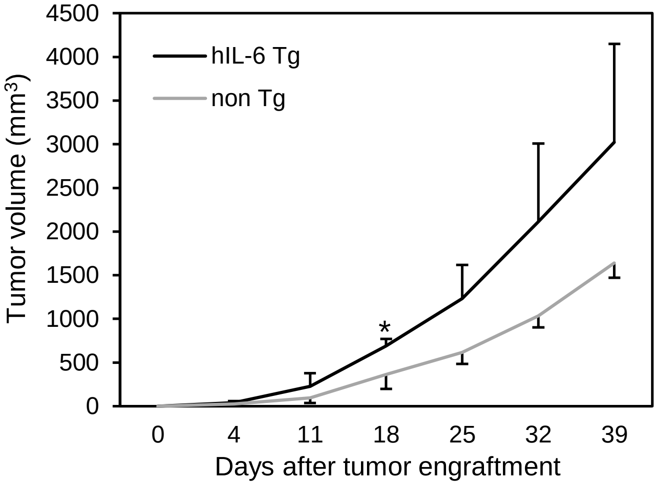

Supplemental Figure 6. Tumor growth in non HSC-transplanted NOG-hIL-6 Tg (n=3) or -non-Tg (n=3) mice. (*p<0.05)
